# Increased BMI favors weaning in patients with chronic intestinal failure due to short bowel syndrome: a retrospective cohort study in Italy

**DOI:** 10.3389/fnut.2025.1672572

**Published:** 2025-11-06

**Authors:** Fabio Dario Merlo, Palle Bekker Jeppesen, Umberto Aimasso, Fabio Bioletto, Marta Ossola, Valentina Ponzo, Ilaria Goitre, Marta Palermo, Elisa Olimpio, Stefano Silveri, Simona Bo

**Affiliations:** 1 Clinical Nutrition Unit, “Città della Salute e della Scienza Hospital”, Torino, Italy; 2 Department of Intestinal Failure and Liver Diseases, Rigshospitalet & Department of Clinical Medicine, University of Copenhagen, Copenhagen, Denmark; 3 Department of Medical Sciences, University of Torino, Torino, Italy

**Keywords:** short bowel syndrome, chronic intestinal failure, parenteral nutrition, body composition, body mass index

## Abstract

**Background:**

A great heterogeneity exists among patients with chronic intestinal failure even with the same intestinal circuit. Weaning from parenteral support depends on intestinal adaptation, remnant bowel length, and functional capacity. The present study aimed to assess if pre-existent nutritional reserves would predict the possibility of enteral autonomy.

**Methods:**

This retrospective observational study evaluated the incidence of weaning off parenteral support in adult patients with chronic intestinal failure due to short bowel syndrome from an Italian referral center. Multivariable models, considering mortality as a competing risk, identified predictors of weaning.

**Results:**

Out of 251 patients, 116 (46.2%) died without being weaned and 76 (30.3%) were weaned off. The latter showed increased residual small bowel length, more frequently the colon-in-continuity and the ileocecal valve, lower age, higher weight and BMI (25.3 ± 5.6 *vs* 20.9 ± 3.2 kg/m^2^) at parenteral support starting. In a multivariable competing risk model, age [sub-distribution hazard ratio (SHR) 0.82; 95%CI 0.71–0.95], small bowel length (SHR = 1.11; 1.06–1.15), type 2 (SHR = 2.63; 1.37–5.02) and type 3 short bowel syndrome (SHR = 6.85; 3.45–13.60), and BMI at enrolment (SHR = 1.11; 1.06–1.15) were significantly associated with weaning off. Body composition by bioelectrical impedance was assessed in a subgroup (*n* = 147). Patients who weaned displayed increased intracellular water as total water percentage, phase angle and muscle mass index. At multivariable analyses, % intracellular water was a significant predictor of weaning (SHR = 1.06; 1.03–1.09).

**Conclusion:**

Patients with chronic intestinal failure due to short bowel syndrome with increased BMI and a healthier body composition were more likely to be weaned off parenteral nutrition.

## 1 Introduction

Chronic intestinal failure (CIF) is a rare but severe condition defined by the inability of the gut to maintain adequate absorption of nutrients and fluids, requiring long-term parenteral support (PS) to sustain life (1). In adults, the leading cause of CIF is short bowel syndrome (SBS), which results from extensive gut resection (2). Dependence on PS greatly impairs quality of life, carries risks of life-threatening complications, and represents a major burden for both patients and healthcare systems, making weaning from PS a critical therapeutic goal (1, 2). Weaning from PS depends on multiple factors, including intestinal adaptation, remnant bowel length, and residual functional capacity (3, 4). In particular, greater residual small bowel length, greater residual colon length, presence of the ileocecal valve, and type of intestinal anatomy have been reported as the most robust predictors of weaning off in adults with CIF (5–8). However, great heterogeneity exists among patients with CIF due to SBS (CIF-SBS) even with the same intestinal circuit. Other factors may be implicated in the weaning processes, such as compensatory hyperphagia, age, coexisting intestinal diseases, reconstructive surgery, pharmacological treatments, above all, trophic agents, as well as blood markers, such as plasma citrulline and apolipoprotein B48 (9–12). Nutritional and metabolic reserves at baseline have rarely been considered as potential determinants of enteral autonomy. From a theoretical point of view, higher body mass index (BMI) may imply a metabolic reserve to support the energy balance during the catabolic stress of intestinal adaptation, improving the patient’s resilience and ability to transition toward enteral autonomy. However, BMI is a crude measure: it does not distinguish lean mass from adiposity, and higher values often reflect increased fat mass, which may be associated with inflammation, insulin resistance, and hepatobiliary disease in PS-dependent patients (13–15). Evidence on this topic is scarce, limited to a few reports suggesting that pre-resection BMI > 35 kg/m^2^ may double the likelihood of PS weaning within 12 months of SBS onset (15), but no study has comprehensively assessed the role of BMI and body composition at the time of PS initiation.

Our Intestinal Failure Unit is a national tertiary referral center for CIF, with one of the largest cohorts of adult patients with CIF-SBS in Italy. This setting provides a well-documented, long-term cohort that accurately represents the clinical challenges of CIF management in our country. However, the issue of weaning from parenteral support in CIF-SBS extends beyond our center. It constitutes a persistent clinical challenge, imposes a major burden on patients, and generates considerable healthcare costs (1, 2), underscoring the clinical relevance and scientific urgency of investigating predictors of weaning in CIF-SBS patients.

Therefore, the aim of the present retrospective cohort study was to evaluate if BMI and body composition at the time of PS starting would predict the possibility of weaning in patients with CIF due to SBS.

## 2 Materials and methods

### 2.1 Patients

All the adult patients with CIF who were treated at the Intestinal Failure Unit of the “Città della Salute e della Scienza” Hospital of Torino from 1st January 1985 to 31st December 2024 were assessed for enrolment.

The inclusion criteria were the need for PS >3 months, age ≥18 years, a diagnosis of SBS confirmed by both surgical reports and radiological examination, exclusion criteria were active neoplastic disease and/or being under antineoplastic treatment within the previous 5 years, inability to give informed consent, critically ill patients, treatment with glucagon-like-peptide 2 agonists (because of their beneficial effects on intestinal absorptive function and PN weaning) and reconstructive surgery.

The database of the Intestinal Failure Unit contains information about all patients with CIF since 1985; it is based on primary data collected by the physicians of the Unit and is continuously updated with prospectively collected clinical data. The follow-up lasted from the beginning of treatment at our center until weaning, or death or the end of observation on 31st December 2024, whichever came first.

Patients with SBS were transferred to the hospital ward of the Intestinal Failure Unit within 7–10 days after surgery; PS was initiated during the surgical hospitalization. BIVA measurements were performed at the time of transfer, reasonably after post-surgical fluid shifts and within the first 7–10 days of parenteral support (median 8 days). BIVA measurements were performed once per patient.

All participants were treated with anti-secretory drugs (proton pump inhibitors) and antidiarrheal medication (loperamide). Patients received personalised fluid and nutritional support in consideration of the individualised needs, according to their body weight, 24-h urine and ostomy output, oral caloric intakes, and laboratory exams. Shared and standardised procedures related to the management and monitoring of patients according to international guidelines are implemented by a team of trained physicians and registered dieticians (4, 16).

All patients were given a diet restricted in fiber and lactose, with an individualised energy amount, tailored to the individual needs. Home PS (HPS) was administered by an intermittent schedule, at night for average 10–16 h/day. Handling of central venous catheters was performed according to international standards by specialized nurses through domiciliary visits (17). Medical follow-up visits were scheduled around every 1–3 months, according to individual need. Management of acute and chronic complications due either to HPS or CIF, as well as periodic follow-up and centralized laboratory and radiological exams were performed according to international standards (17).

### 2.2 Data collection

Information of all patients with CIF were collected since 1985, from PS starting until weaning or death in the unit database. SBS was diagnosed with a remnant small bowel length ≤200 cm (18). Anatomical types of SBS were defined as: type 1 (end-jejunostomy), type 2 (jejunocolonic anastomosis), type 3 (jejunoileal anastomosis). The residual intestinal length was measured by a barium or Gastrografin follow-through examination performed by the same trained radiologist. Colon length was reported according to the method of Cummings in case of a colon in continuity (19). Data relative to the number of previous abdominal surgeries prior to the surgery leading to SBS were also collected.

Weaning from PS was defined as the complete discontinuation of fluid and/or nutrition infusion with maintenance of metabolic and clinical stability by the patients until the end of follow-up. Metabolic and clinical stability was considered in the presence of consistently adequate urine output (at least 1,000 mL/day), stable body weight (i.e., change in body weight <2%), normal vital parameters and acid–base balance, serum metabolic variables and serum/urinary electrolyte values within range of normality (i.e., serum sodium 135–145 mmol/L, serum potassium 3.5–5 mmol/L, serum chloride 97–107 mmol/L, serum magnesium 0.77–1.03 mmol/L, serum phosphorus 0.81–1.45 mmol/L; urinary sodium 75–200 mmol/day), and absence of nutritional deficiencies.

Body weight and height were measured with the patients wearing light clothes and no shoes by a mechanical column scale (SECA model 711) and a Stadiometer (SECA 220 measuring rod, Hamburg, Germany), at fasting in the morning, after urination, and emptying of the stoma bags.

Body composition was assessed by single-frequency bioelectrical impedance (BIA 101, Akern, Pisa Italy). The exam was carried out under standardised conditions, with the patients fasting for at least 8 h, lightly dressed, after voiding, without heavy physical activity during the preceding 12 h. Data relative to the impedance raw values (resistance, R and reactance, Xc) were normalized for subject’s height (R/h and Xc/h, Ohm/m) to eliminate conductor length effect, and the bioelectrical impedance vector analysis (BIVA) software was employed. Phase angle (PhA), total water (as percentage of body weight) extra-cellular water (ECW) and intracellular water (ICW) (both as a percentage of total water), as well as fat mass (FM) index (FM/height^2^) and muscle mass (MM) index (MM/height^2^) were analysed.

BIVA analysis has been routinely performed on all patients at the start of PS since 2010. Therefore, data was available for a subset of participants.

### 2.3 Ethical issues

At the time of PN starting, patients gave their written informed consent to the processing of their data. The study was approved by the local Ethics Committee (protocol number CS2/740/2018), and all the procedures were in accordance with the principles of the Declaration of Helsinki.

### 2.4 Statistical analyses

Data about weaning from HPS were analysed through cumulative incidence functions, obtained using an Aalen-Johansen estimator (20) considering mortality as a competing risk; the analysis was stratified according to BMI class and tested for differences according to the log-rank test (21). The association between prognostic factors and the outcome of interest was first assessed with univariate competing risk Cox regressions, with the estimation of sub-distribution hazard ratios (SHRs) (22). Afterward, clinical predictors were assessed for inclusion in a multivariable model using a stepwise backward selection, using *p* < 0.05 as the stopping rule; weight was excluded due to collinearity with BMI. After obtaining a multivariable model considering only clinical predictors, BIVA parameters were also evaluated, and their role as independent predictors was again assessed using a stepwise backward selection approach. Model calibration was evaluated by estimating the calibration slope, obtained by refitting a Fine–Gray model with the original model’s linear predictor as the sole covariate. Internal validation was performed using 1,000 bootstrap resamples to assess optimism in the calibration slope. As this was a retrospective cohort study including all consecutive eligible patients treated at our referral center between 1985 and 2024, no ‘*a priori*’ sample size calculation was performed. The sample size was determined by case availability on a pragmatic base and was sufficient to ensure an adequate events-per-variable ratio for the multivariable analyses.

## 3 Results

### 3.1 Patients

**Figure 1:**
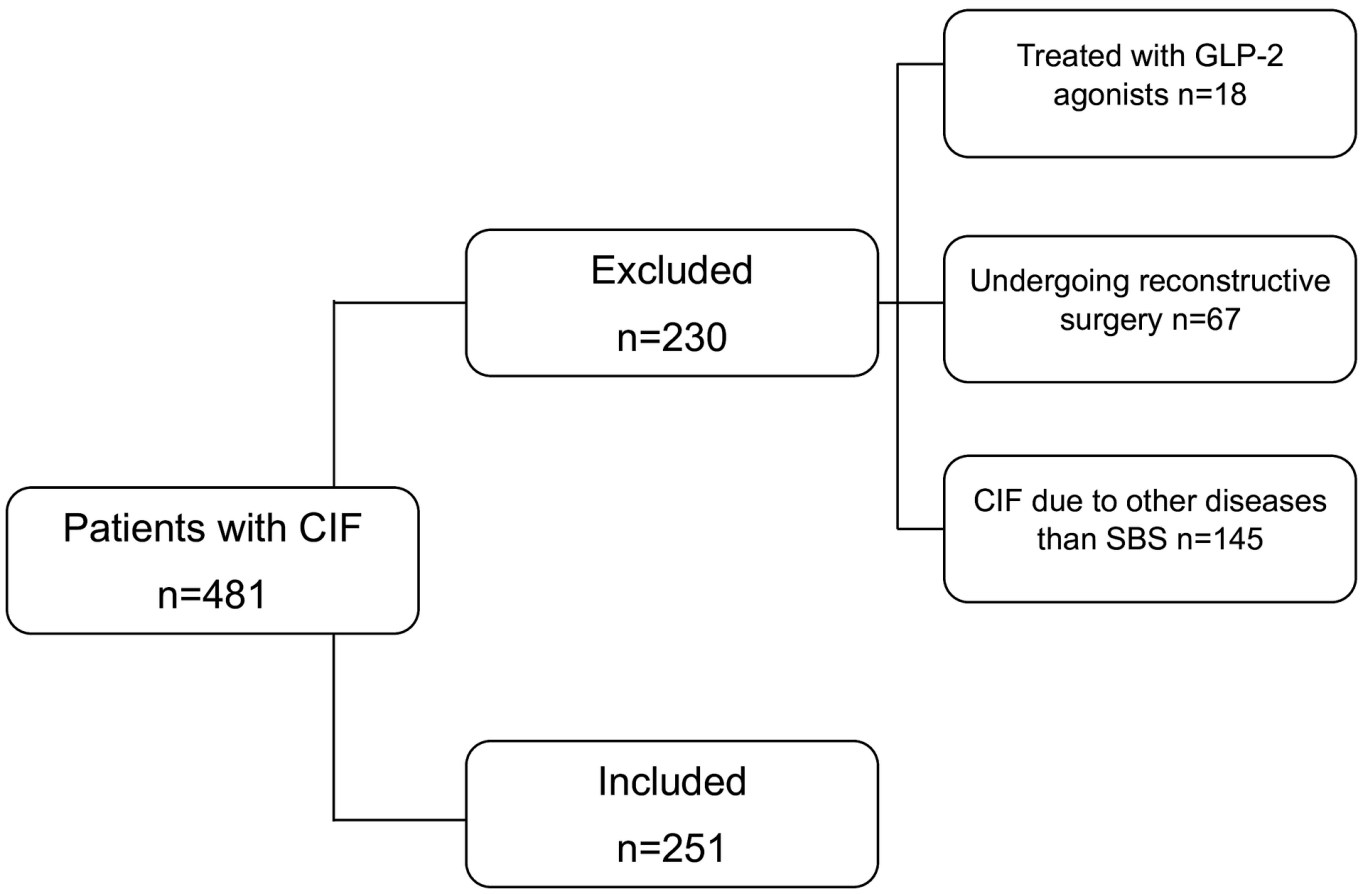
Flow of the study.

All consecutive adult patients with CIF-SBS treated at our referral center between 1985 and 2024 were assessed for enrolment. Out of 481 patients, 251 met the inclusion criteria (Figure 1). Their characteristics at enrolment are reported in Table 1.

**Table 1:** Clinical characteristics at enrolment of the whole group of participants (left) and stratified by weaning occurrence during follow-up (right).

Variables	All	Weaned NO	Weaned YES	*p*
Number	251	175	76	
Small bowel length (cm)	89.4 ± 54.2	81.5 ± 53.2	107.6 ± 52.6	<0.001
Ileocecal valve (%)	61 (24.3)	27 (15.4)	34 (44.7)	<0.001
Classification by anatomy group				
Type 1 (%)	95 (37.8)	78 (44.6)	17 (22.4)	
Type 2 (%)	95 (37.8)	70 (40.0)	25 (32.9)	
Type 3 (%)	61 (24.3)	27 (15.4)	34 (44.7)	<0.001
Underlying disease				
Crohn disease (%)	47 (18.7)	34 (19.4)	13 (17.1)	
Radiation enteritis (%)	24 (9.6)	16 (9.1)	8 (10.5)	
Surgery due to cancer (%)	67 (26.7)	45 (25.7)	22 (29.0)	
Mesenteric ischemia (%)	79 (31.5)	55 (31.4)	24 (31.6)	
Fibro-adhesive peritonitis (%)	20 (8.0)	13 (7.4)	7 (9.2)	
Other causes (%)	14 (5.6)	12 (6.9)	2 (2.6)	0.795
Number of previous abdominal surgeries* (%)				
0	95 (37.8)	66 (37.7)	29 (38.2)	
1	45 (17.9)	30 (17.1)	15 (19.7)	
2	53 (21.1)	38 (21.7)	15 (19.7)	
≥3	58 (23.1)	41 (23.4)	17 (22.4)	0.955
Males (%)	122 (48.6)	87 (49.7)	35 (46.1)	0.594
Age of PS starting	61.2 ± 15.1	62.5 ± 15.6	58.2 ± 13.6	0.038
Weight (kg)	59.4 ± 13.4	56.0 ± 10.6	67.3 ± 15.8	<0.001
BMI (kg/m^2^)	22.2 ± 4.5	20.9 ± 3.2	25.3 ± 5.6	<0.001

*Number of abdominal surgeries prior to the surgery leading to SBS.

*p*-values obtained by Student’s *t*-test or chi-square test.

The mean age at PS starting was 61.2 ± 15.1, with a slight predominance of females (51.4%). The most frequent causes underlying SBS were mesenteric ischemia, surgical resection due to cancer, and Crohn diseases. Weaning was defined by durable metabolic and clinical stability (i.e., the presence of consistently adequate urine output, stable body weight, normal vital parameters and acid–base balance, serum metabolic variables and serum/urinary electrolyte within range of normality, and absence of nutritional deficiencies). Out of enrolled participants: 116 (46.2%) died without being weaned and 76 (30.3%) were weaned from PS during follow-up. On December 3rd, 2024, 63 patients were alive and weaned, while 59 were still on HPS.

### 3.2 Characteristics of patients by baseline BMI

At baseline, 60 (23.9%) patients were overweight or had obesity (BMI ≥ 25 kg/m^2^) and 191 (76.1%) had a BMI < 25 kg/m^2^. Patients with overweight/obesity showed a significantly higher small bowel length than individuals with BMI < 25 kg/m^2^ (105.4 ± 53.5 *vs* 84.4 ± 53.6, *p* = 0.008) and different prevalence of causes, being prevalence of Crohn disease 10.0 and 21.5%, and of mesenteric ischemia 45.0% and 27.2%, respectively, in patients with BMI ≥ 25 kg/m^2^ and BMI < 25 kg/m^2^ (*p* = 0.10) (Supplementary Table 1). No differences were observed in the presence of the ileocecal valve, SBS classification, or number of previous abdominal surgeries. At enrolment, 147 (56.8%) participants were assessed by BIVA. Patients with higher BMI had lower percentage of total body water and proportion of extracellular water, and greater proportion of intracellular water. They also exhibited significantly higher muscle mass index and fat mass index than patients with BMI < 25 kg/m^2^.

### 3.3 Characteristics of patients by weaning occurrence during the follow-up

**Figure 2:**
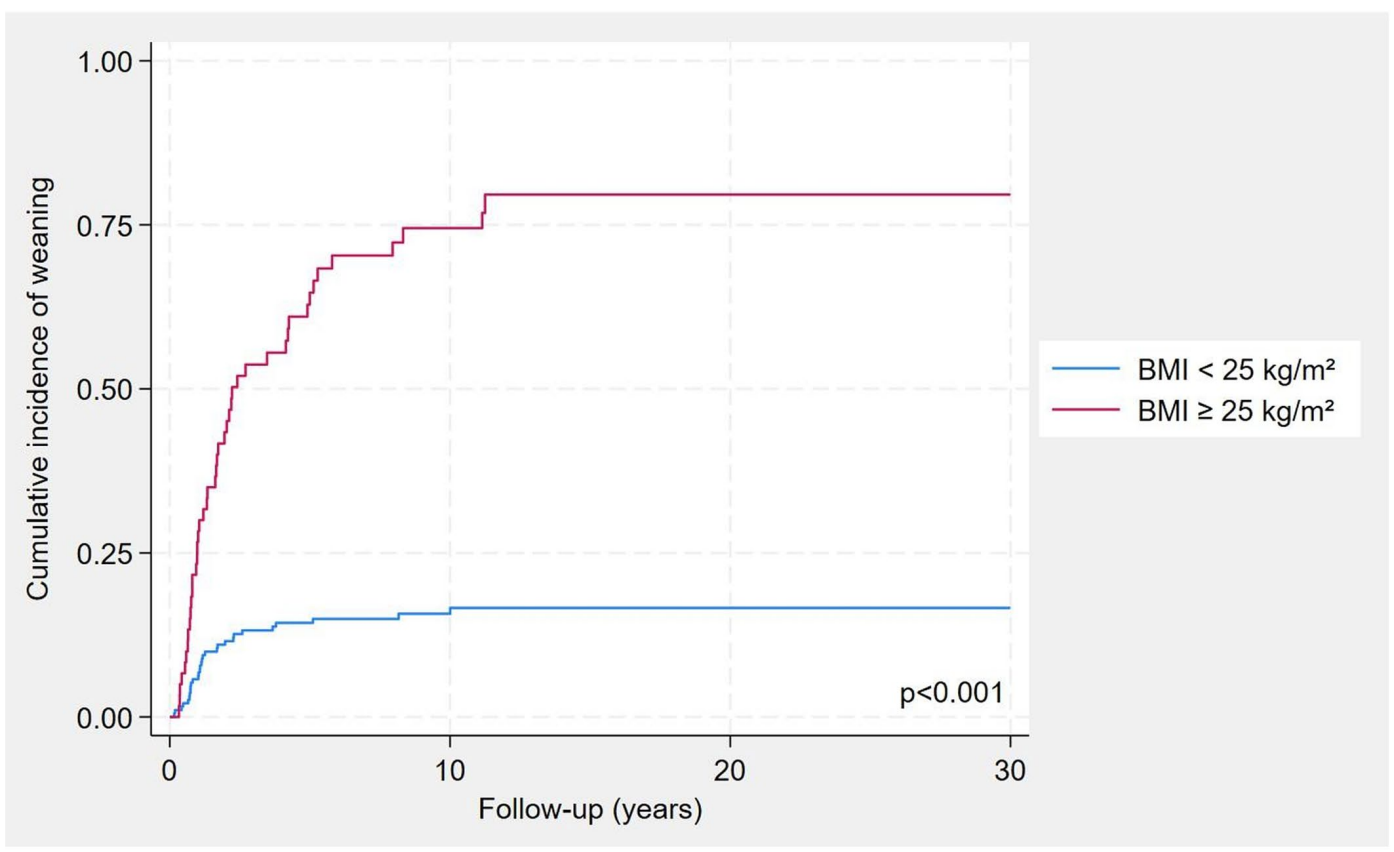
Cumulative incidence of weaning by BMI classes, considering mortality as a competing risk.

Patients who were weaned from HPS during the follow-up where characterised by increased length of the residual small bowel, lower age at PS starting, higher weight and BMI at baseline (Table 1). As expected, patients who weaned had more frequently a type 3 SBS and presence of the ileocecal valve. In Figure 2, the cumulative incidence of weaning was shown by classes of BMI and was significantly higher in patients with BMI ≥ 25 kg/m^2^ compared to those with BMI < 25 kg/m^2^ (log-rank *p* < 0.001).

#### 3.3.1 BIVA variables by weaning occurrence during the follow-up

BIVA measurements were performed at a median of 8 days after initiation of PS. Out of 147 participants submitted to BIVA analysis, 42 were successfully weaned during follow-up and 105 were not (Table 2). Patients who were successfully weaned showed significantly lower resistance values and higher phase angle compared with those not weaned. They also presented lower percentage of total body water and proportion of extracellular water, but greater proportion of intracellular water, muscle mass and fat mass indices. Cumulative incidence of weaning in the overall cohort for BMI strata (<25 vs. ≥ 25 kg/m^2^) and in the BIVA subset for ICW/TBW tertiles are shown in Supplementary Table 2.

**Table 2:** BIVA values at enrolment of the whole group of participants (left) and stratified by weaning occurrence during follow-up (right).

	All	Weaned NO	Weaned YES	*p*
Number	147	105	42	
R (ohm)	559.7 ± 127.5	573.8 ± 135.0	524.5 ± 99.3	0.034
R/h (ohm/m)	345.2 ± 83.0	354.0 ± 87.2	323.2 ± 67.3	0.042
Xc (ohm)	41.9 ± 13.5	41.0 ± 13.7	44.1 ± 12.9	0.222
Xc/h (ohm/m)	25.8 ± 8.4	25.3 ± 8.5	27.0 ± 8.0	0.246
PhA (°)	4.42 ± 1.22	4.29 ± 1.28	4.74 ± 0.96	0.042
TBW (%)	58.0 ± 10.6	60.1 ± 10.2	53.0 ± 10.0	<0.001
ECW/TBW (%)	54.7 ± 9.0	56.3 ± 8.8	50.7 ± 8.3	<0.001
ICW/TBW (%)	45.3 ± 9.0	43.7 ± 8.8	49.3 ± 8.3	<0.001
Muscle mass index (kg/m^2^)	9.3 ± 3.0	8.5 ± 2.3	11.3 ± 3.6	<0.001
Fat mass index (kg/m^2^)	6.6 ± 3.4	6.2 ± 3.0	7.6 ± 4.1	0.022

*p*-values obtained by Student’s *t*-test.

### 3.4 Clinical characteristics by anatomical types of SBS

At enrolment, significant differences were observed across SBS anatomical types (Supplementary Table 3). As expected, patients with type 1 SBS had a markedly longer remnant small bowel length and higher number of previous abdominal surgeries compared with those with type 2 or type 3 anatomy. The distribution of underlying disease also varied significantly, with mesenteric ischemia predominating in type 2 patients, whereas Crohn’s disease was more common in type 1 and cancer-related surgery in type 1 and 3. By contrast, no significant differences were found between anatomical types in terms of sex distribution, age at PS initiation, body weight, or BMI.

### 3.5 BIVA variables by anatomical types of SBS

Body composition parameters assessed by BIVA did not differ significantly across SBS types. Resistance, reactance, phase angle, and water distribution (percentage TBW, ECW/TBW, ICW/TBW) were comparable among the three groups (Supplementary Table 4). Similarly, no significant differences were observed in muscle mass or fat mass indices, although a non-significant trend toward higher muscle mass index was noted in type 3-SBS patients.

### 3.6 Competing risk analysis

At univariate Cox-regression analysis, considering mortality as a competing risk, age, TBW% and ECW/TBW% (inversely), and small bowel length, type 3 SBS, weight, BMI at enrolment, ICW/TBW%, MM index and FM index (directly) were associated with the probability of weaning (Table 3, left).

**Table 3:** Predictors of weaning from HPS in CIF-SBS patients, considering mortality as a competing risk.

Parameter	Univariate analysis		Multivariable analysis considering clinical parameters		Multivariable analysis considering clinical and BIVA parameters	
	SHR (95%CI)	*p*-value	SHR (95%CI)	*p*-value	SHR (95%CI)	*p*-value
Age at baseline (per 10-year increase)	0.86 (0.76–0.98)	0.021	0.82 (0.71–0.95)	0.007	–	–
Sex						
Female	1 (ref)		–		–	
Male	0.89 (0.57–1.39)	0.604	–	–	–	–
Underlying disease						
IBD	1 (ref)		–		–	
Radiation enteritis	1.17 (0.48–2.87)	0.727	–	–	–	–
Surgical resection	1.18 (0.59–2.35)	0.644	–	–	–	–
Mesenteric ischemia	1.01 (0.52–1.95)	0.985	–	–	–	–
Fibroadhesive peritonitis	1.29 (0.49–3.35)	0.605	–	–	–	–
Other	0.91 (0.26–3.19)	0.882	–	–	–	–
Previous surgeries						
0	1 (ref)		–		–	
1	1.22 (0.65–2.28)	0.538	–	–	–	–
2	1.00 (0.54–1.85)	0.993	–	–	–	–
≥3	1.12 (0.62–2.04)	0.702	–	–	–	–
SBS type						
Type 1	1 (ref)		1 (ref)		1 (ref)	
Type 2	1.44 (0.78–2.65)	0.243	2.63 (1.37–5.02)	0.004	3.75 (1.52–9.25)	0.004
Type 3	4.08 (2.26–7.37)	<0.001	6.85 (3.45–13.60)	<0.001	10.85 (4.47–26.32)	<0.001
Small bowel length (per 10-cm increase)	1.08 (1.04–1.12)	<0.001	1.11 (1.06–1.15)	<0.001	1.08 (1.02–1.14)	0.006
Weight (per 1 kg increase)	1.04 (1.03–1.06)	<0.001	–	–	–	–
BMI (per 1 kg/m^2^ increase)	1.12 (1.08–1.18)	<0.001	1.11 (1.06–1.15)	<0.001	1.13 (1.08–1.18)	<0.001
Rz (per 1 Ohm increase)	1.00 (0.99–1.00)	0.006	N/A	N/A	–	–
Xc (per 1 Ohm increase)	1.01 (0.99–1.03)	0.290	N/A	N/A	–	–
PhA (per 1 unit increase)	1.20 (0.99–1.46)	0.068	N/A	N/A	–	–
TBW% (per 1% increase)	0.94 (0.92–0.97)	<0.001	N/A	N/A	–	–
ECW/TBW% (per 1% increase)	0.95 (0.92–0.98)	0.002	N/A	N/A	–	–
ICW/TBW% (per 1% increase)	1.05 (1.02–1.09)	0.002	N/A	N/A	1.06 (1.03–1.09)	<0.001
MM index (per 1 kg/m^2^ increase)	1.23 (1.15–1.32)	<0.001	N/A	N/A	–	–
FM index (per 1 kg/m^2^ increase)	1.07 (1.02–1.13)	0.009	N/A	N/A	–	–

*Number of abdominal surgeries prior to the surgery leading to SBS.

N/A, not analysed.

*p* from univariate analysis obtained by competing risk Cox regressions according to the Fine & Gray model.

In a multivariable model, age (inversely) and small bowel length, type 2 and type 3 SBS, BMI at enrolment (directly) were significantly associated with weaning off from HPS. When expanding this model by considering BIVA variables, the same associations as above were found, except for age, that was excluded being no longer statistically significant. On the other hand, ICW as a percent of TBW was a significant predictor of weaning, with an approximately 6% increased probability for each 1% of ICW increase (Table 3, right).

Internal validation using 1,000 bootstrap resamples showed minimal optimism in the calibration slope (mean −0.02, SD 0.11), indicating excellent internal calibration and no evidence of overfitting.

## 4 Discussion

In a large cohort of patients with CIF-SBS, BMI values at the time of PS commencement were directly associated with the probability of weaning in a multivariable model, considering mortality as a competing risk, after taking into account the residual bowel length and the anatomical circuit. In a subset of patients, for whom BIVA analysis was performed, ICW as a percent of TBW, reflecting body cell mass and muscle mass, was a significant predictor of enteral autonomy in the same model.

### 4.1 The role of the gut in the weaning process

In adult patients with SBS, a small bowel length of <100 cm has been reported as highly predictive of progression to CIF requiring long-term parenteral support (5). The presence of the terminal ileum with ileocecal valve and/or the colon in continuity increases the probability of weaning by improving absorption of water, electrolytes, and nutrients (particularly carbohydrates), enhancing hyperphagia, contributing to the secretion of pancreatic and biliary juices and gastrointestinal hormones, and supporting the activity of the colonic microbiota, which produces short-chain fatty acids (SCFAs) with beneficial metabolic effects (1, 5, 23–26). Nevertheless, heterogeneity in PS dependence exists among patients with similar anatomical circuits, and anatomy alone predicts weaning in only about 55% of cases (9). Digestive adaptation starts early after gut resection and continues over the following 1–5 years, involving structural changes, enhanced residual gut function, and slowed intestinal transit, processes mediated by hormones, trophic factors, nutrients, and the microbiota (1, 3). Additional variability may arise from host-related factors affecting absorption and adaptation, individual nutritional requirements, and external exposures such as luminal stimuli, specific nutrients, trophic agents, reconstructive surgery, or microbiota-modulating therapies (3, 9, 10, 16). Underlying disease may also play a role, since disease trajectories can impact long-term nutritional reserves (27). Finally, age has been proposed as a prognostic factor, but literature remains controversial (4, 7, 28).

In our cohort, each 10-cm increase in small bowel length was associated with an approximately 10% higher probability of weaning, confirming the strong influence of remnant bowel length. Patients with a colon in continuity displayed more than a twofold higher probability of acquiring enteral autonomy compared with those with an end-jejunostomy or end-ileostomy (type 1 anatomy). As in previous reports, we observed that cumulative incidence of weaning was maximal within the first 5 years after resection and then reached a plateau (Figure 2). However, despite these associations, we also found substantial heterogeneity: patients with the same anatomical type displayed variable outcomes, underscoring that anatomy alone is insufficient to explain PS dependence.

We further explored other potential predictors. Arterial mesenteric ischemia, the most common cause of SBS in European cohorts, typically affects older adults and may present in individuals with higher baseline BMI (7, 28, 29). Individuals who suffer an acute catastrophic event, such as intestinal infarction, often start in better nutritional condition than those with chronic illnesses, repeated surgeries, or multiple health challenges over time who typically experience a gradual decline in their energy reserves (27). Although this condition might be expected to affect adaptation, we found no significant differences in weaning rates. This finding may be attributable to the inclusion of only those patients who have received at least 3 months of PS, as individuals with acute conditions might not require prolonged nutritional support.

On the other hand, age at PS initiation was inversely related to the probability of weaning in our cohort. This aligns with some prior studies suggesting lower likelihood of weaning with increasing age (4), but contrasts with others reporting no such association (7, 28). These results highlight the complexity of the weaning process and reinforce the need for multifactorial prognostic models that integrate anatomical, clinical, and host-related factors.

### 4.2 The role of body composition in the weaning process

In our cohort, BMI at PS initiation was independently associated with the probability of weaning, with an ~11% increase in odds per 1 kg/m^2^ increase. Patients who reached enteral autonomy displayed healthier body composition, including both higher phase angle -suggesting cell membrane integrity and better cellular health- and enhanced proportion of intracellular water and muscle mass index, all consistent with preserved nutritional status and lower risk of sarcopenia (30). These findings support that body composition, rather than BMI *per se*, is a more reliable predictor of weaning.

Previous studies have suggested a relationship between BMI and the probability of weaning from PS. A BMI of 25–30 kg/m^2^ has been associated with an 80% higher probability of 1-year weaning, and a BMI ≥ 30 kg/m^2^ with a twofold higher likelihood in large international cohorts of CIF patients (31). Similarly, pre-surgical BMI > 35 kg/m^2^ was reported to predict more than a twofold greater chance of weaning within 1 year than lower BMI values (58% *vs* 21%) though with increased hepatobiliary complications (15). Other studies, however, found no such association, possibly because BMI values were recorded during PS rather than at its start, raising the possibility of confounding by artificial nutrition (26).

Increased BMI may reflect better nutritional status, but this is not the case in SBS patients, where it is an unreliable marker (13, 14). Indeed, normal BMI can mask malnutrition due to altered body composition, fluid shifts, or compensatory hyperphagia. Furthermore, malnutrition and sarcopenia remain prevalent even in SBS patients with normal or elevated BMI (13, 14, 32–34). These findings highlight the importance of assessing body composition directly. Reduced fat-free mass and muscle depletion are common during or after PS, and muscle mass is linked to hyperphagia, frailty, and overall prognosis (35–37). Moreover, parenteral nutrition itself has been shown to alter body composition by increasing fat stores (13). The gut microbiota can influence body composition and energy balance by enhancing nutrient absorption, promoting intestinal adaptation, producing SCFAs, and modulating metabolism and inflammation (16, 38–42). In addition, obesity-associated microbiota and prior metabolic exposures (“metabolic memory”) may exert lasting effects on energy extraction and metabolism, even after weight loss or changes in nutritional status (43–47).

In our cohort, the percentage of intracellular water emerged as an independent predictor of weaning. A higher %ICW reflects better cellular hydration, metabolic stability, and overall cell health, indicating the absence of ongoing catabolism or fluid-electrolyte disturbances. This interpretation is consistent with evidence from studies in elderly and dialysis populations, where increased ICW has been associated with improved clinical outcomes (48, 49).

Finally, whether a healthier body composition is linked to favorable anatomical conditions rather than being a truly predictive factor of weaning should be considered. In our cohort, patients with different SBS anatomical types displayed a significant heterogeneity in underlying disease and remnant small bowel length, reflecting the different etiologies and surgical trajectories leading to CIF. In contrast to the marked variability in anatomy and disease background, body composition parameters were largely comparable across the three SBS anatomical types. This finding suggests that factors beyond anatomical configuration—such as individual adaptation capacity, and host-related characteristics—may play a critical role in shaping long-term nutritional condition and weaning potential.

### 4.3 Clinical implications

Our findings indicate that nutritional and metabolic reserves are critical determinants of intestinal adaptation, complementing established anatomical predictors. In this context, BMI and %ICW emerge as practical tools for risk stratification and for guiding individualized weaning strategies. Integrating metabolic and body composition assessment into multidisciplinary care pathways for patients with CIF-SBS could therefore strengthen clinical decision-making, support individualized follow-up intensity, and help set realistic expectations with patients and carers regarding the probability of achieving enteral autonomy. Although BIVA provides valuable insights into body composition and cellular hydration, its availability is limited in many centers. In such settings, simple measures such as BMI combined with handgrip strength or standard bioimpedance parameters may serve as pragmatic surrogates for risk stratification.

### 4.4 Limitations and strengths

The retrospective nature of the study should be recognized as a limitation, even if we have taken care of considering potential bias in the weaning process by excluding patients on treatment with glucagon-like-peptide 2 agonists or submitted to reconstructive surgery. Furthermore, to minimize selection bias, our study included all consecutive eligible adult patients with chronic intestinal failure due to short bowel syndrome treated at our referral center between 1985 and 2024. Therefore, the cohort is representative of our patient population, and not subject to arbitrary selection. Although this was a single-center study, in Italy only a few specialized centers manage chronic intestinal failure, and all follow international guidelines for parenteral support. Small units may use slightly different practices, but the number of patients treated outside referral centers is very low, making our findings broadly generalizable. The lack of the assessment of intestinal energy absorption capacity may compromise the validity of the definition of metabolic stability of participants. Nevertheless, the routine use of metabolic balance studies, which are recognized as the gold standard for determining absorptive capacity, is complex, time-consuming, high-cost, and usually employed for research purposes only. However, ours is a referral center with 40 years of experience in managing CIF-SBS patients; the close follow-up, a standardized approach according to international guidelines, and the centralized diagnostic procedures have allowed us to acquire solid expertise in the clinical evaluation of these patients. Anthropometric measurements were not collected before surgery. However, BIVA assessments were performed at an early stage after initiation of parenteral support, once post-surgical fluid shifts had stabilized. Assessment of the body composition was made in a subset of patients by BIVA. We acknowledge that this may introduce potential selection bias, as patients treated before 2010 were excluded from body composition analysis. Nevertheless, the associations identified are biologically plausible and consistent with existing evidence, which strengthens the validity of our findings. Furthermore, we have carefully addressed potential confounders by adjusting all analyses for major clinical and anatomical predictors of weaning in multivariable competing risk models. BIVA measurement is quick, noninvasive, requires limited operator training and maintenance, ensures comparability of data during the long period of enrolment of our patients. Specific predictive equations for SBS patients are lacking, and therefore equations used to estimate MM are not specifically validated for patients with SBS, who often display marked fluid alterations. Indeed, BIVA was supported by literature in SBS patients (50), and more complex techniques, such as dual-energy X-ray absorptiometry (DEXA), are not free of limitations in those individuals (51). A further limitation is the lack of gut microbiome data, which may influence nutrient absorption and intestinal adaptation independently of BMI or %ICW. Finally, although we adjusted for SBS type and residual bowel length, residual confounding by underlying etiology (e.g., mesenteric ischemia, Crohn’s disease, cancer) cannot be fully excluded. Different disease trajectories may have shaped long-term nutritional reserves through a “metabolic memory” effect, as discussed, and this should be considered when interpreting our findings.

The strengths of the present study were the large cohort studied, the use of appropriate statistical tools to deal with competing risks and prevent outcome overestimation, the centralization of the laboratory analyses, and the standardization of the diagnostic approach and treatment by a multidisciplinary team with a long-lasting expertise in the management of these patients.

## 5 Conclusion

Patients with a healthier body composition at the start of parenteral support maintained an advantage over time and are weaned more frequently. In addition to intestinal anatomy, individual factors related to metabolic memory, or the gut microbiome might be involved. These findings require further confirmation and could have interesting implications for clinical practice.

## Data Availability

The datasets presented in this article are not readily available. Because of participant confidentiality and privacy concerns, data cannot be shared publicly and requests to data access must be submitted in writing. Requests to access the datasets should be directed to simona.bo@unito.it.
