# Supplementary material for: Increased BMI favors weaning in patients with chronic intestinal failure due to short bowel syndrome: a retrospective cohort study in Italy
Source: Front Nutr. 2025 Nov 6;12:1672572. doi: 10.3389/fnut.2025.1672572 (PMC12631374; doi:10.3389/fnut.2025.1672572)
Supplement: Supplementary file 1 [file Table_1.docx]

**Supplementary Table 1. Clinical characteristics at enrolment of participants stratified by BMI at PS commencement**

|  | **BMI <25 kg/m^2^** | **BMI ≥25 kg/m^2^** | **P** |
| --- | --- | --- | --- |
| Number | 191 | 60 |  |
| Small bowel length (cm) | 84.4±53.6 | 105.4±53.5 | 0.008 |
| Ileocecal valve (%) | 44 (23.0) | 17 (28.3) | 0.404 |
| Classification |  |  |  |
| Type 1 (%) | 73 (38.2) | 22 (36.7) |  |
| Type 2 (%) | 74 (38.7) | 21 (35.0) |  |
| Type 3 (%) | 44 (23.0) | 17 (28.3) | 0.696 |
| Underlying disease |  |  |  |
| Crohn disease (%) | 41 (21.5) | 6 (10.0) |  |
| Radiation enteritis (%) | 18 (9.4) | 6 (10.0) |  |
| Surgery due to cancer (%) | 51 (26.7) | 16 (26.7) |  |
| Mesenteric ischemia (%) | 52 (27.2) | 27 (45.0) |  |
| Fibro-adhesive peritonitis (%) | 17 (8.9) | 3 (5.0) |  |
| Other causes (%) | 12 (6.3) | 2 (3.3) | 0.095 |
| Number of previous abdominal surgeries* (%) |  |  |  |
| 0 | 72 (37.7) | 23 (38.3) |  |
| 1 | 35 (18.3) | 10 (16.7) |  |
| 2 | 41 (21,5) | 12 (20.0) |  |
| ≥3 | 43 (22.5) | 15 (25.0) | 0.970 |
| Males (%) | 94 (49.2) | 28 (46.7) | 0.731 |
| Age of PS starting | 60.7±16.1 | 62.7±11.3 | 0.388 |
| Weight (kg) | 54.7±9.9 | 74.6±11.9 | <0.001 |
| BMI (kg/m^2^) | 20.3±2.8 | 28.2±4.0 | <0.001 |
| Number | 106 | 41 |  |
| R (ohm) | 584.5±130.9 | 495.5±92.2 | <0.001 |
| R/h (ohm/m) | 360.8±85.4 | 304.9±60.8 | <0.001 |
| Xc (ohm) | 42.7±14.0 | 39.8±12.2 | 0.235 |
| Xc/h (ohm/m) | 26.3±8.8 | 24.3±7.1 | 0.188 |
| PhA (°) | 4.38±1.29 | 4.51±1.00 | 0.574 |
| TBW (%) | 60.6±10.2 | 51.5±8.7 | <0.001 |
| ECW/TBW (%) | 55.7±8.9 | 52.2±8.9 | 0.036 |
| ICW/TBW (%) | 44.4±8.9 | 47.8±8.9 | <0.001 |
| Muscle mass index (kg/m^2^) | 8.6±2.3 | 11.1±3,7 | <0.001 |
| Fat mass index (kg/m^2^) | 5.7±2.5 | 9.0±4.2 | <0.001 |

* Number of abdominal surgeries prior to the surgery leading to SBS; p-values obtained by Student’s t-test or chi-square test

**Supplementary Table 2. Cumulative incidence of weaning over time by BMI strata and tertiles of % ICW/TBW**

|  | **Cumulative incidence of weaning over time** | | | | |
| --- | --- | --- | --- | --- | --- |
|  | **1 year** | **3 years** | **5 years** | **10 years** | **20 years** |
| Overall cohort | 10.8% | 22.9% | 26.5% | 29.9% | 32.0% |
| Stratified by BMI |  |  |  |  |  |
| BMI < 25 kg/m^2^ | 5.8% | 13.2% | 14.4% | 15.7% | 16.6% |
| BMI ≥ 25 kg/m^2^ | 26.7% | 53.7% | 64.5% | 73.6% | 77.2% |
| Stratified by % ICW/TBW tertiles |  |  |  |  |  |
| First tertile | 4.1% | 12.3% | 17.1% | 17.1% | 17.1% |
| Second tertile | 2.0% | 14.7% | 20.3% | 25.8% | 31.4% |
| Third tertile | 18.4% | 28.7% | 33.2% | 43.9% | 48.3% |

**Supplementary Table 3. Clinical characteristics at enrolment according to SBS classification**

|  | **Type 1** | **Type 2** | **Type 3** | **P** |
| --- | --- | --- | --- | --- |
| Number | 95 | 95 | 61 |  |
| Small bowel length (cm) | 109.6±53.6 | 76.7±52.9 | 77.9±48.3 | <0.001 |
| Underlying disease |  |  |  |  |
| Crohn disease (%) | 24 (25.3) | 19 (20.0) | 4 (6.6) |  |
| Radiation enteritis (%) | 9 (9.5) | 9 (9.5) | 6 (9.8) |  |
| Surgery due to cancer (%) | 33 (34.7) | 14 (14.7) | 20 (32.8) |  |
| Mesenteric ischemia (%) | 19 (20.0) | 41 (43.2) | 19 (31.2) |  |
| Fibro-adhesive peritonitis (%) | 6 (6.3) | 7 (7.4) | 7 (11.5) |  |
| Other causes (%) | 4 (4.2) | 5 (5.3) | 5 (8.2) | 0.004 |
| Number of previous abdominal surgeries* (%) |  |  |  |  |
| 0 | 22 (23.2) | 46 (48.4) | 27 (44.3) |  |
| 1 | 17 (17.9) | 17 (17.9) | 11 (18.0) |  |
| 2 | 26 (27.4) | 16 (16.8) | 11 (18.0) |  |
| ≥3 | 30 (31.6) | 16 (16.8) | 12 (19.7) | 0.011 |
| Males (%) | 48 (50.5) | 47 (49.5) | 27 (44.3) | 0.730 |
| Age of PS starting | 62.3±14.8 | 61.5±13.9 | 58.8±17.3 | 0.358 |
| Weight (kg) | 60.7±14.7 | 57.1±11.4 | 61.0±14.1 | 0.101 |
| BMI (kg/m^2^) | 22.4±4.6 | 21.7±3.7 | 22.7±5.5 | 0.303 |

* Number of abdominal surgeries prior to the surgery leading to SBS; p-values obtained by Student’s t-test or chi-square test

**Supplementary Table 4. Body composition parameters by BIVA according to SBS classification**

|  | **Type 1** | **Type 2** | **Type 3** | **P** |
| --- | --- | --- | --- | --- |
| Number | 64 | 48 | 35 |  |
| R (ohm) | 553.5±127.6 | 571.6±134.3 | 554.5±119.9 | 0.733 |
| R/h (ohm/m) | 339.9±85.4 | 356.4±87.1 | 339.5±73.0 | 0.524 |
| Xc (ohm) | 41.9±13.9 | 41.1±12.2 | 43.0±14.8 | 0.829 |
| Xc/h (ohm/m) | 25.6±8.6 | 25.7±7.8 | 26.3±9.0 | 0.926 |
| PhA (°) | 4.44±1.13 | 4.45±1.47 | 4.32±0.97 | 0.860 |
| TBW (%) | 58.7±10.2 | 59.6±11.1 | 54.8±10.4 | 0.101 |
| ECW/TBW (%) | 54.9±8.4 | 54.3±10.6 | 54.9±7.9 | 0.922 |
| ICW/TBW (%) | 45.2±8.4 | 45.7±10.6 | 45.1±7.9 | 0.930 |
| Muscle mass index (kg/m^2^) | 9.3±2.4 | 8.6±2,9 | 10.1±3,7 | 0.071 |
| Fat mass index (kg/m^2^) | 6.9±3.9 | 6.4±2.9 | 6.2±3.1 | 0.523 |

p-values obtained by Student’s t-test
